# East Suffolk Hospital, Ipswich

**Published:** 1912-06-01

**Authors:** 


### East Suffolk Hospital, Ipswich.

EXTENSION OF THE OUT-PATIENT DEPARTMENT.

A Special Court of Governors met in the Board-room of the Hospital, Ipswich, on May 22, to decide whether they should or should not take the bold step of embarking upon further enlargements at this critical time in the history of the voluntary system. The meeting was presided over by Dr. J. H. Bartlet, president of the hospital, who has been connected with the staff of this hospital over forty-three years. Mr. F. Messent, chairman of the board of management, Mr. E. A. Oliver, chairman of the workmen's committee, Dr. R. W. Brogden, Dr. J. F. C. Hossack, Admiral Pelham Aldrich, and Major H. M. Barnes were also present.

The scheme presented was the result of a report drawn up in collaboration with the medical staff by a special committee consisting of Mr. John D. Cobbold and Mr. Herbert Mason, two members of the weekly board, and Mr. Arthur Griffiths, the secretary, after they had visited several hospitals in the Metropolis and the provinces. The scheme consisted of (*a*) an *x*-ray department, including electric baths and massage for out-patients (the only existing apparatus being in the main block, from which the out-patient block is entirely detached, and this apparatus is only sufficient for in-patient work); (*b*) an operating theatre for casualties and minor operations, with an anæsthetising room and recovery room; and (*c*) surgeries for casualties. The estimated cost of the scheme was given as £3,450, £800 of which is being taken from revenue, £350—the estimated cost of appliances—is being given by Mr. Cobbold, and the remaining £2,300 was proposed to be withdrawn from capital.

Mr. Mason, in proposing the adoption of the scheme, read a letter which had been received from a firm employing a large number of hands foreshadowing a falling off of support both from the employers and employees as a result of the Insurance Act. Mr. Mason claimed that it was not for the management of the hospital to shirk its responsibilities to the sick for fear of the result of any legislation; the hospital work was to a large extent special work for which special apparatus and special provision had to be made, and which could not be obtained other than in such an institution. In his opinion it was both unlikely and undesirable that the grand principle of the voluntary hospital system of this country should be abolished in favour of State support and State control, and he asked the employers of labour whether it would not cost them more to maintain the hospitals by taxation than under the present system, which was infinitely better for the patient. The resolution, which was seconded by the President, who associated himself with all the previous speaker had said, was carried unanimously.

It will be remembered, as previously reported in The Hospital, that structural alterations and additions were completed in 1910 at a cost of £20,000. Since then open-air balconies have been added to the children's wing and a porter's lodge erected at the main entrance, and at the present moment an addition is being made to the nurses' home, adding sixteen bedrooms and other apartments. Thus far the board of management have been able to carry out these extensions without serious declension of their invested capital or any loss, we understand, on the income and expenditure account.

